# Do preterm infants’ retinas like bovine colostrum? A randomized controlled trial

**DOI:** 10.1186/s13052-024-01781-z

**Published:** 2024-10-19

**Authors:** Marwa Mohamed Farag, Mohamed Alaa Eldin Hassan Thabet, Islam SH Ahmed, Nesrine Fathi Hanafi, Walaa Samy Elsawy, Eman Shabban Mohamed

**Affiliations:** 1 Pediatrics and Neonatology, Pediatric Department, Alexandria University Faculty of Medicine, Alexandria University, Alexandria, Egypt; 2 Ophthalmology Department, Alexandria University Faculty of Medicine, Alexandria University, Alexandria, Egypt; 3 Medical Microbiology and Immunology Department, Alexandria University Faculty of Medicine, Alexandria, Egypt

**Keywords:** Retinopathy of prematurity, Bovine colostrum, Liposomal delivery system, Weight gain

## Abstract

**Background:**

Bovine colostrum (BC) with liposomal delivery system (LDS) is a promising supplement to premature infant formula in absence of mother own milk. We propose that BC with LDS can target multiple etiological factors that threaten the developing retina, making premature infant less liable for retinopathy of prematurity (ROP). The aim of this study was to evaluate the effect of BC with LDS in the prevention of ROP.

**Methods:**

This was a single center, randomized, controlled trial. Two hundred and eleven preterm infants of gestational age ≤ 32weeks were admitted to the NICU of Alexandria University Children Hospital, Egypt, and randomly allocated into either non-BC group (*n* = 105) or BC group (*n* = 106). Patients in BC group received 3.5 ml /kg/day of BC for 14 days. All patients were monitored for development of ROP, anemia, late onset sepsis (LOS), bronchopulmonary dysplasia (BPD), periventricular leukomalacia (PVL) and necrotizing enterocolitis (NEC), in addition to growth assessment. Multivariate binary logistic regression analysis was performed to determine factors predicting ROP development.

**Results:**

Compared with the non-BC group, BC group was associated with a significantly lower incidence of ROP (5/100 vs. 16/100, respectively) with a p-value of 0.033. The administration of BC significantly decreased serum C- reactive protein (CRP) level and increased weight on day-14 of the study in comparison with the CRP level and birthweight at the beginning of study, with Cohen’s D= -0.184, D = -2.246, respectively. Patients with suspected sepsis were significantly less in BC than non-BC group, *p* = 0.004. Patients with BC had significantly higher hemoglobin level on day-14 than non-BC-group, with median (IQR) 12.2 (11.0–13.9) and 11.7 (10.5–12.9), respectively. BC intake is one of factors that decreased development of ROP in univariate analysis. Nevertheless, weight gain and birth weight were the most significant factors affecting ROP development in multivariate-regression model.

**Conclusion:**

BC may reduce the incidence of ROP in preterm neonates aged ≤ 32 weeks. This might be due to keeping better Hb level and growth rate, as well as anti-inflammatory properties through its ability to decrease CRP level.

**Trial registration:**

This work was registered on 06/13/2022 in clinicaltrial.gov with ID no.: NCT05438680 and URL:https://classic.clinicaltrials.gov/ct2/show/NCT05438680?term=NCT05438680&draw=2&rank=1.

**Supplementary Information:**

The online version contains supplementary material available at 10.1186/s13052-024-01781-z.

## Background

Fresh mother own milk (MOM) is considered the optimal milk diet for healthy term infants and infant formula (IF), indeed, is less efficient in stimulating gut maturation, feeding tolerance, resistance against necrotizing enterocolitis and neurodevelopment [1]. MOM availability in low- and middle-income countries (LMIC) might be hampered even if the mother is willing to breastfeed her baby, due to the higher incidence of neonatal intensive care unit (NICU) admissions for preterm or low birth weight newborns, as well as to major problems for the mothers making them unable to visit or send milk to her baby regularly, due to financial or other specific constrains.

Donor milk (DM) may also be suboptimal because of low bioactivity of pasteurized, thawed human milk from other mothers obtained during late lactation [2]. No differences were found in necrotizing enterocolitis (NEC) and late onset sepsis (LOS) incidence when MOM was supplemented with either DM or IF in two large clinical trials [3, 4]. Further, some countries are unable to use DM because of religious and ethical issues.

Bovine colostrum (BC) is the first milk from cows produced within the first 24 h after parturition. BC is closely adapted to provide nutrition, immunization and microbial protection due to its high levels of proteins that include bioactive components potentially active across different mammalian species [5]. 

Retinopathy of prematurity (ROP) is a vasculo-proliferative disease of retina and a leading cause of visual impairment. It has two-phases, delay in retinal vascular growth (phase I) and hypoxia- induced release of factors stimulating neovascularization, including VEGF (phase II). IGF-1 has a critical role in normal vascular development [6]. Poor postnatal weight gain or weight loss is associated with low IGF-1 that predisposes for ROP.

Prematurity related complications commonly have multifactorial pathogenesis. We believe that BC in absence of MOM might help in antagonizing many aspects of those pathogenetic factors. ROP screening of patients receiving BC can uncover this potentially useful treatment option, and might be a starting point for searching the effect of BC on other prematurity related complications.

The primary objective of this work was to test whether BC with LDS is effective in preventing ROP in preterm infants with GA ≤ 32 weeks (regardless of birth weight), if they were stable enough to have feeding in the first three days of life and were planned to receive IF in the absence of MOM. Secondary objectives were to study the impacts of BC with LDS on anthropometry and especially on weight gain, anemia and need for blood transfusion, feeding tolerance, and stage II and III of NECas well as its anti-inflammatory effect indirectly evaluated through CRP levels analysis.

## Methods

### Study design, setting, and participants

**Fig. 1:**
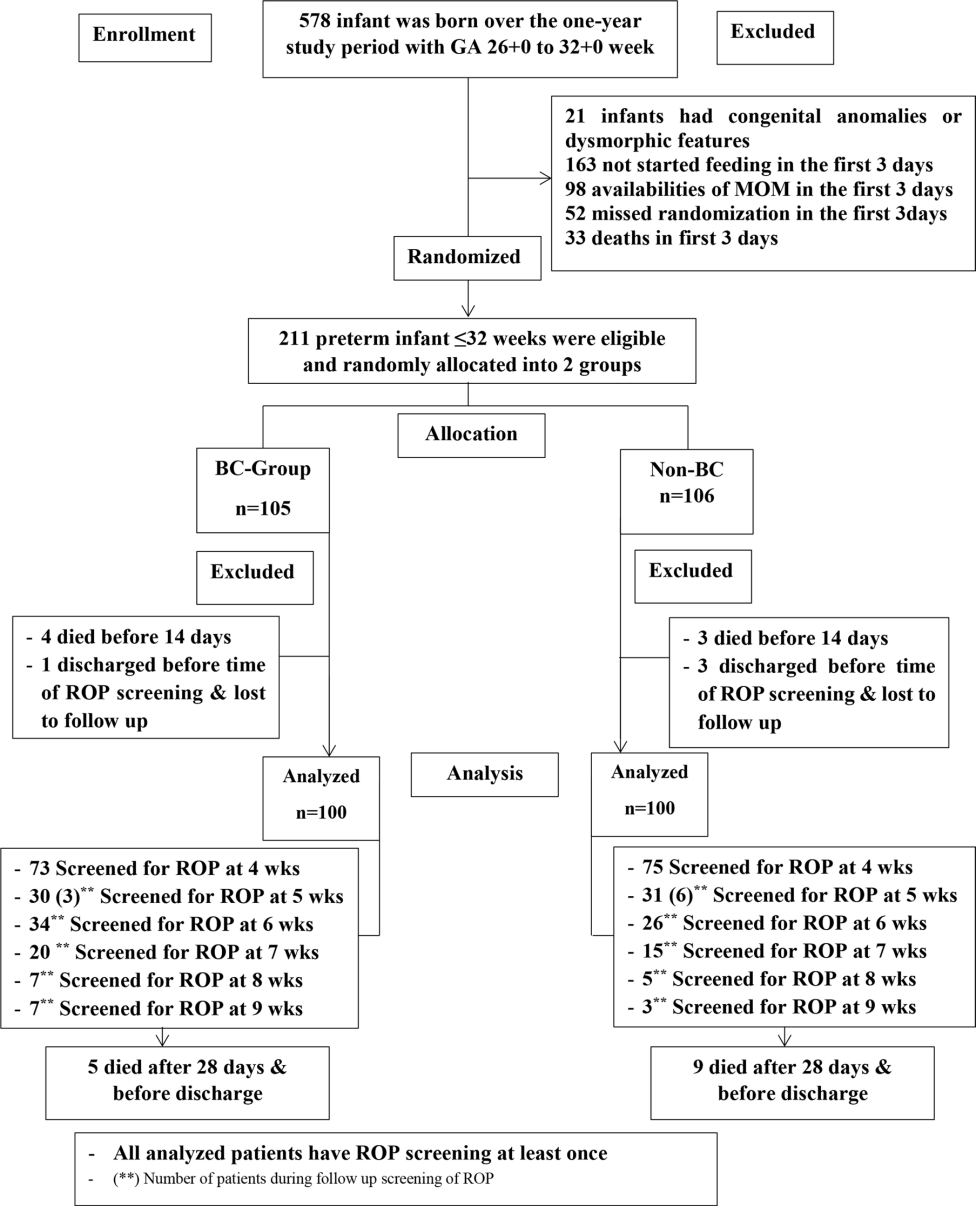
Consort flow chart for patients

This study was a two-armed randomized controlled trial conducted at the NICU of Alexandria University Maternity Hospital (AUMH). AUMH is a large tertiary referral center that provides service to infants and mothers coming from 4 governorates in North Egypt, with annual NICU admissions of 1400–2200 patients. The majority of these subjects are premature infants < 37 weeks. Infants born in AUMH with GA 26 + 0 to 32 + 0 weeks and admitted to the NICU, regardless their weight, were enrolled within 72 h of birth at a study site, if they were eligible and written informed consent was obtained from parents. Patients with major congenital anomalies, clinically suspected TORCH infections, having MOM and/or mothers intended to breast feed her infant and patients who did not start feeding in the first 72 h were excluded from the study before randomization. Among the latter there were those with evidence of perinatal asphyxia, severe IUGR, and those newborns who were not expected to survive > 1 week after admissions. If patients died before completing 14 days or MOM became available, they also were excluded from the analysis. Only patients who had ROP screen at 28 days or more (*n* = 100 in non-BC and *n* = 100 in BC-groups) were included in the analysis (per-case protocol), Fig. 1.

### Ethics-approval and consents

Study protocol has been approved on 20th May 2021 by the Research Ethics Committee of Alexandria Faculty of Medicine. Written informed consents were obtained from parents of all newborns who participated in the study for participation and publication of anonymous patients’ data.

### Randomization and blinding

Randomization was applied using sealed envelope technique with a ratio of 1:1 using random permuted blocks of 4 and 6 size, prepared by a collaborator not involved in the study. Patients were randomized into two groups, BC group and non -BC group. BC sachets were reconstructed by a trained nurse assistant who prepared the infant formula for all patients daily. All clinical staff members, except the ophthalmologist and the data evaluator, were not blinded to the intervention groups because of technical reasons.

### Nutrition protocol and intervention

Daily fluid, energy and protein were given in enteral and parenteral nutrition according to the practical guidelines for preterm nutrition, ESPGHAN and CSPEN guidelines [7–9]. Patients had trophic feeds 10 ml/kg of IF (PreNAN formula, Nestle, Amsterdam, Netherland ^®^) with the amount of BC subtracted from it. All infants enrolled in the study had their MOM unavailable and started feeding in the first 72 h. Then, feeds were advanced by 10–20 ml per kg daily to reach full feeds of 180 ml per kg. After the intervention period (14 days), all patients were fed with IF. Infant formula provides 0.0232 g protein and 0.80 Kcal/ml. No routine use of probiotics or antifungal prophylaxis for the studied patients have been applied. Eligible patients were randomized into 2 groups: BC group with patients receiving BC with LDS from reconstituted Baby-Steps powder in dose of 3.5 ml (1 ml colostrum)/kg/day and non-BC group who received only IF.

Powder of Baby-Steps (5 g)- sachet with 300 mg IgG (NIG Nutritionals, Auckland, New Zealand ^®^) was reconstructed in 50 ml lukewarm water. The powder is a lactose - and fat-reduced, high protein product and includes a number of growth factors (e.g. insulin-like growth factors I and II), antimicrobial factors (e.g. immunoglobulins), cytokines, enzymes, hormones, and other components. It contains LDS, a phospholipid bilayer naturally present in goat’s milk, used to increase the bioavailability of any bioactive ingredient encapsulated within it from 10 to15 times, reducing thus the administered dose. Without LDS, regular colostrum has the drawbacks of poor absorption, low bioavailability and being susceptible for the digestive enzymes. LDS helped to solve all these problems and decrease the dose [10]. The dose used was 3.5 ml (1 ml Colostrum)/kg/day of reconstituted powder. The protein and energy contents are 0.04 g/ml, 0.45 kcal/ml, respectively. Both nutritional compositions of IF and BC powder are present in the supplementary material, S-Table 8 and S-Table 9.

Weight in grams was measured and recorded daily from admission to 14 days of life using a calibrated digital scale. The head circumference and the body length were measured weekly using firm plastic tape with local standards.

### Primary outcome: evaluation of patients by fundus examination

ROP screening was done by a single ophthalmologist starting at least at 28 days of life by indirect ophthalmoscopy and thirty diopter lenses, and the infants were followed up at an interval determined by the ophthalmologist according to the degree of ROP severity until achievement of full retinal vascularization, or achieving 40 weeks post-natal age. ROP was classified according to ICROP3 [11]. It classifies ROP based on the severity (stage), antero-posterior location (zone), and presence or absence of plus disease or progression to ROP-requiring-treatment (anti-VEGF). Severe ROP requiring treatment according to the early treatment ROP {ETROP} criteria include: any ROP in zone I with plus disease; stage III ROP in zone I with or without plus disease; or stage II + or III + in zone II [12]. We considered the worst stage and zone in the analysis of the results.

### Secondary outcomes and safety

Follow up of the whole clinical course and outcomes were reported for all patients. Secondary outcomes included various growth parameters (including weight, weight gain, head circumference and length), and feeding intolerance (defined as bilious gastric residuals, vomiting, or gastric residuals more than half of the feeding volume in the first 14 days of life). Days to reach full feeds (DTFF), were also reported in all patients. Stage II or III of NEC was defined according to modified Bell’s criteria [13], while anemia requiring packed red cells (PRBCs) transfusion was defined according to Kasat-et-al.criteria for blood transfusion [14]. Inflammation was indicated by CRP. Regarding sepsis, patients were classified into no, possible, probable, and culture- proven sepsis [15]. In addition, sepsis was classified according to the onset into early and late onset sepsis (EOS and LOS), before and after day 3 of life, respectively. The clinical course and prematurity related morbidities were recorded, including duration of ventilation, need for inotropes, duration of hospital stay and fate, bronchopulmonary dysplasia (BPD, patients were in need of oxygen supplementation +/- ventilatory support for ≥ 28 days postnatal age or ≥ 36 weeks postmenstrual age) [16], intraventricular hemorrhage (IVH) and periventricular leukomalacia (PVL), diagnosed by ultrasonography, and cow milk allergy (clinical diagnosis includes one or more of the following manifestations that respond to the elimination of cow’s milk: diarrhea, apnea, rectal bleeding, or an acute manifestation resembling NEC, shock, or sepsis) [17]. Biochemistry and hematology laboratory investigations were done on postnatal day 1 and 14 to identify the benefits and harms of the intervention. First, we gathered initial laboratory information to see whether the two groups had an equal chance of experiencing problems. Then, after 14 days, we checked to see if there were any laboratory abnormalities at the end of the BC treatment. There were no notable deviations observed in the two groups.

### Sample size calculation

Sample size was calculated using Power Analysis and Sample Size Software (PASS 2020) “NCSS, LLC. Kaysville, Utah, USA, ncss.com/software/pass”. We estimated the sample size concerning 5 important study variables (weight gain, feeding tolerance, need of blood transfusion due to anemia, C-RP levels, and rate of developing retinopathy of prematurity), and considered the highest minimal sample size accordingly. Based on previous published data [18, 19], 170 was the minimal total hypothesized sample size considered to evaluate the efficacy of BC administration as a prophylaxis to decrease the incidence and the occurrence of ROP in preterm < 32 weeks regardless of their birthweight. We utilized an effect size of 10%, a significance level of 5% and a power of 80% using Chi square test [20]. The conducted research’s sample size was 200 patients,100 for each group; BC group depicted 20% lower incidence rate of suspected sepsis compared to non-BC-group (*p* = 0.004). Thus, post-hoc power analysis of the studied sample population has been estimated to be **90%**.

### Statistical methods

Data were fed to the computer and analyzed using IBM SPSS software package version 20.0. The Kolmogorov–Smirnov test was used to verify the normality of distribution. Qualitative data were described using number and percent. Quantitative data were described using range (minimum and maximum), mean, and standard deviation, median and interquartile range (IQR). Significance of the obtained results was judged at the 5% level. Student t-test, Monte Carlo test, Chi-square test, and Fisher Exact test were used for comparison between the three groups regarding different variables. Cohen’s d was used to measure the size of the difference between D1 and D14, regarding weight and CRP level in the two groups. We interpreted the effect sizes as small (d = 0.2), medium (d = 0.5), and large (d = 0.8) based on benchmarks suggested by Cohen − (1988). Multivariate binary logistic regression analysis was performed to determine factors predicting ROP development. Backward stepwise regression was done, with potential confounders retained in the base models if they changed the effect of the explanatory variable (BC) both clinically and statistically (changing the crude OR by ≥ 10%). Moreover, all the included variables were statistically significant (*P* < 0.05) in the univariate analysis. In case of collinearity between two or more variables, only one variable was retained in the model. Collinearity was found between birth weight and GA, as well as between tachycardia and hypoactivity. Therefore, one independent variable for each of them were included in the regression model.

## Results

### Enrollment, baseline characteristics and in-hospital course

In total, 578 infants were born in AUMH over the one-year study period with GA 26 + 0 to 32 + 0 weeks, and 211 infants fulfilled the inclusion criteria and were randomized to BC (*n* = 105) and non-BC group, (*n* = 106) in the study period (Fig. 1). Two hundred patients completed the study and were included in the analysis, 100 in each group. Baseline characteristics, resuscitation data and maternal risk factors of infants in both groups were shown in Table 1. Male gender represented 55/100 of BC-group and 53/100 of non-BC group with p-value 0.777. The mean ± SD values for GA in weeks for BC-group and non-BC group were 30 ± 1 and 31 ± 1, respectively, p-value 0.609. While, mean values for birth weight in grams were 1184 ± 211 for BC group and 1231 ± 216 for non-BC group, p-value 0.12. One-minute Apgar score showed significant difference in both study groups, while this difference disappeared at 5 min.

S-Table 2 shows no significant differences between the two study groups regarding need for ventilation, duration of ventilation, inotropic support, deaths and duration of hospital stay.

**Table 1:** Comparison between the two studied groups as regards demographic and perinatal data

	Groups	Test of sig.	Test of sig	P
	BC	Non-BC		
**Maternal age in years**				
Min. – Max.	18–36	18–42	U= 3971	0.012^*^
Mean ± SD.	25 ± 4	26 ± 5		
Median (IQR)	24 (22–28)	26 (23–30)		
**Residency**				
Rural	51 (51.0%)	56 (56.0%)	χ^2^= 0.502	0.478
Urban	49 (49.0%)	44 (44.0%)		
**Sex**				
Male	55 (55.0%)	53 (53.0%)	χ^2^= 0.081	0.777
Female	45 (45.0%)	47 (47.0%)		
**GA in weeks**				
Min. – Max.	27–32	27–32	U = 4796.5	0.609
Mean ± SD.	30 ± 1	31 ± 1		
Median (IQR)	31 (30–32)	31 (30–32)		
**BWT in grams**				
Min. – Max.	760–1670	670–1800	t= -1.541	0.125
Mean ± SD.	1184 ± 211	1231 ± 216		
Median (IQR)	1185 (1000–1350)	1230 (1065–1400)		
**Antenatal steroids**				
Complete	39 (39.0%)	29 (29.0%)	χ^2^= 2.230	0.328
Incomplete	35 (35.0%)	41 (41.0%)		
No	26 (26.0%)	30 (30.0%)		
**Maternal sepsis**	53 (53.0%)	56 (56.0%)	χ^2^= 0.181	0.670
**Hypertension**	14 (14.0%)	38 (38.0%)	χ^2^= 14.969	< 0.001^*^
**PET**	17 (17.0%)	32 (32.0%)	χ^2^= 6.082	0.014^*^
**DM**	7 (7.0%)	9 (9.0%)	χ^2^= 0.272	0.602
**MOD**				
CS	80 (80.0%)	72 (72.0%)	χ^2^= 1.754	0.185
NVD	20 (20.0%)	28 (28.0%)		
**Resuscitation**				
Initial steps	71 (71.0%)	79 (79.0%)	χ^2^= 4.207	^MC^p = 0.113
PPV (Ambu)	23 (23.0%)	20 (20.0%)		
PPV (ETT)	6 (6.0%)	1 (1.0%)		
**APGAR at 1 min**				
<7	64	44	χ^2^= 8.052	0.005*
≥7	36	56		
**APGAR at 5 min**				
< 7	26	18	χ^2^= 1.865	0.172
≥ 7	74	82		

**χ**^**2**^: Chi square test **U**: Mann Whitney test **t**: Student t-test

p: p value for comparing between the studied groups *: Statistically significant at *p* ≤ 0.05

MOD: Mode of delivery GA: Gestational age BWT: Birth weight DM: Diabetes mellitus PET: Preeclampsia

### Primary outcome (ROP) and risk factors related to ROP

S-Table-5 demonstrates significant difference between the two study groups regarding different stages of ROP. Stage 0 with no ROP represented the majority of patients in BC group (95/100) and in non-BC group (84/100). Sixteen patients in non-BC group and 5 in the BC- group developed ROP. Patients with ROP stage I and II (ROP-not requiring treatment, Table 2), were more in non-BC group (*n* = 13) in comparison to BC group (*n* = 4). Stage III ROP was more in non-BC group (*n* = 3) than BC-group. However, no significant differences were reported between the groups neither in ROP requiring treatment (Table-2) nor plus/pre-plus diseases development. Zones depict the location of the disease in the retina and vascular maturation. The majority of patients in both groups had zone III involvements. 9/16 patients in non-BC group and 5/5 patients in BC-group have both right and left eyes involvement.

**Table 2:** Comparison between the two groups regarding presence of ROP and treatment requirement

		Groups		Test of significance	P
		B.C	Non-BC		
**Anti-VEGF Injection**	No ROP	95 ^a^(95.0%)	84^b^ (84.0%)	χ^2^=6.44	^MC^*p* = 0.033*
	ROP-non-requiring treatment	4 ^a^(4.0%)	13^b^ (13.0%)		
	ROP-requiring treatment	1^a^ (1.0%)	3^a^ (3.0%)		

**χ**^**2**^: Chi square test **VEGF**: vascular endothelial growth factor **ROP**: retinopathy of prematurity

p: p value for comparing between the studied groups

*: Statistically significant at *p* ≤ 0.05

In each row: different letters are significant

S-Table 6(a, b and C) showed univariate analysis of risk factors for ROP development in the whole study sample. Gestational age, birthweight, weight gain, LOS, apnea, feeding intolerance, tachycardia, hypoactivity, thermal instability, BPD, PVL/IVH, invasive ventilation, blood sample results (complete blood picture, coagulation profile and CRP level), and transfusion needs significantly increase, while intake of BC significantly decreased ROP occurrence.

Table-3 demonstrated backward stepwise regression model using BC as explanatory variable. Both birth weight and weight gain were significantly related to ROP development with OR 0.994 (0.991–0.997) and 0.987 (0.980–0.994), respectively.

**Table 3:** Multivariate Logistic regression analysis for the parameters affecting ROP (final model). Multivariate binary logistic regression analysis was performed to determine factors predicting ROP development. Backward stepwise regression was done, with potential confounders retained in the base models if they changed the effect of the explanatory variable (Bovine Colostrum) both clinically and statistically (changing the crude OR by ≥ 10%). Moreover, all the included variables were statistically significant (*P* < 0.05) in the univariate analysis. In case of collinearity between two or more variables, only one variable was retained in the model. Collinearity was found between (birth weight vs. gestational age) and (tachycardia vs. hypoactivity)

	*P* value	OR	Multivariate	
			95% C.I. for OR	
			Lower	Upper
**Birth weight**	< 0.001^*^	0.994	0.991	0.997
**Hypothermia**	0.098	2.815	0.825	9.601
**weight gain**	< 0.001^*^	0.987	0.980	0.994

Variables in the base model were as follows: Bovine Colostrum, birth weight, weight gain, tachycardia, bronchopulmonary dysplasia, thermal abnormalities (hypothermia) and ventilation

OR: Odd`s ratio C.I: Confidence interval *: Statistically significant at *p* ≤ 0.05

Figure-1 demonstrates timing of the ROP screening. Seventy- three of BC-group and 75 patients of non-BC group were screened for the first time at age of 4 weeks. At 5 weeks postnatal age, 30 patients of BC group were screened for ROP (27 patients were screened for the first time and 3 were screened for the second time). Similarly, at age of 5 weeks, 31 patients of non-BC group were screened (25 patients were screened for the first time and 6 patients were screened for the second time). Thereafter, patients in both groups were examined according to the ophthalmologist’s recommendations.

### Secondary outcomes and safety

#### Growth parameters

**Fig. 2:**
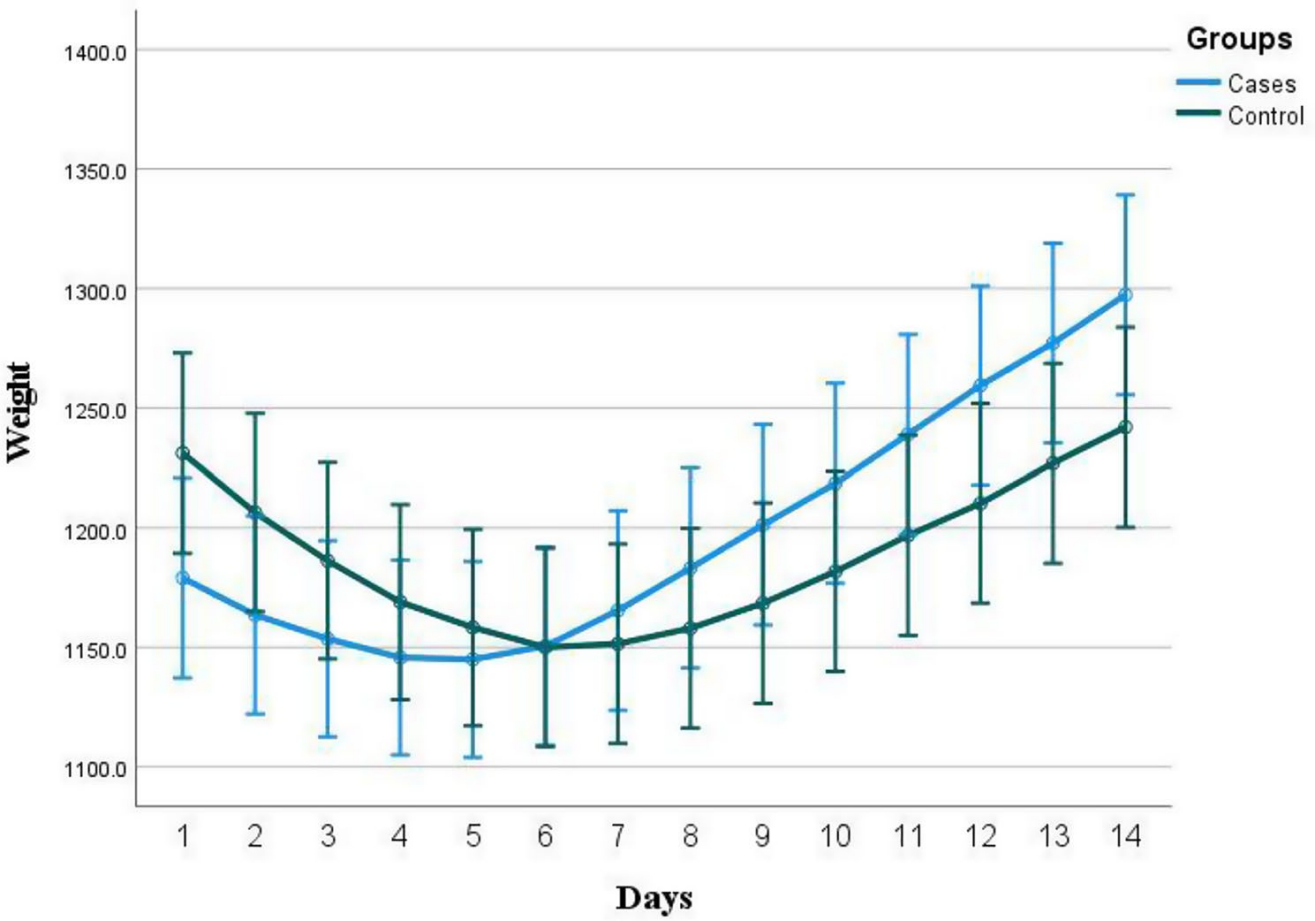
Weight change along the first 14 days of life in the two studied groups, BC group (cases) and non-BC group (control)

S- Table-1 shows no significant difference between the 2 studied groups regarding length and head circumferences. Initially weight measures were higher in the non-BC group with p-value 0.084. However, weight measures at 14 days, at time of ROP screen and at discharge were higher in the BC group, with p-value 0.066, 0.065 and 0.086, respectively. Weight gain in the first 14 days was significantly higher in the BC group *p* < 0.001; Figure-2 shows timeline weight measures in both study groups along the first 14 days of life.

In BC-group, Cohen’s d indicates a large mean difference (effect size, D = -2.246) between D1 and D14 weight. Nevertheless, no significant effect size (Cohen’s d -0.176) was reported for weight in the non-BC group (Table-4).

**Table 4:** Changes in weight and CRP in both BC and control group

	D1	D14	Test of significance	P	Effect size
**Weight in BC group**					
Min. – Max.	760.0–1670.0	825.0–1725.0	t= -22.456	< 0.001^*^	Cohen’s d -2.246
Mean ± SD.	1179.1 ± 208.6	1297.0 ± 207.7			
Median (IQR)	1175.0 (1000.0–1350.0)	1308.5 (1132.5–1465.0)			
**CRP in BC group**					
Min. – Max.	0.1–86.0	0.1–58.0	Z= -2.607	0.009^*^	-0.184
Mean ± SD.	6.4 ± 11.2	4.4 ± 9.2			
Median (IQR)	3.0 (0.5–7.0)	1.5 (0.5–3.0)			
**Weight in non-BC group**					
Min. – Max.	670.0–1800.0	660.0–1840.0	t= -1.762	0.081	Cohen’s d -0.176
Mean ± SD.	1231.2 ± 215.6	1242.1 ± 215.8			
Median (IQR)	1230.0 (1065.0–1400.0)	1247.5 (1092.5–1395.0)			
**CRP in non-BC group**					
Min. – Max.	0.1–62.0	0.1–94.0	Z= -0.379	0.705	-0.027
Mean ± SD.	6.1 ± 9.0	7.1 ± 13.9			
Median (IQR)	3.3 (1.5–6.4)	2.6 (1.2–4.9)			

Z: Wilcoxon signed ranks test t: Paired t-test

p: p value for comparing between two groups *: Statistically significant at *p* ≤ 0.05

#### Feeding intolerance, necrotizing enterocolitis and duration to reach full feeds

Occurrence of feeding intolerance was similar in both study groups. Days to reach full feed were not different in BC- group,10.0 (8.0–12.0) in comparison to non-BC group 11.0 (8.0–13.0) with p-value 0.31. Only two patients in BC group had NEC stage II /III as shown in S-Table 2.

#### Anemia and need for blood transfusion

Hb values were similar in both groups at time of admission, while Hb was significantly decreased in the non-BC group at 14 days, S-Table 3. The need for PRBCs-transfusion, number of transfusions, frequency of sampling and patients with anemia (Hb < 10 g/dl) were not significantly different in both groups. Nevertheless, blood transfusion and number of transfusions were significant factors for ROP in the univariate analysis with OR (95% C.I), 14.757 (1.938–112.376) and 1.487 (1.023–2.163), and p-values of 0.009^*^ and 0.038, respectively, S-Table 6.

#### Sepsis, inflammation and other prematurity related complication

Regarding onset of sepsis, no differences between the study groups were demonstrated in LOS, while EOS was significantly higher in non-BC group. Furthermore, no significant differences in both study groups regarding presence of culture proven and probable sepsis were observed, while there was a significant difference in possible sepsis (clinically diagnosed sepsis), *p* = 0.004*, S-Table 3. LOS was proved to be risk factor for ROP in univariate analysis, S-table-6b.

Except for hypoactivity, no differences were demonstrated between both groups regarding clinical findings including, apnea, tachycardia, abnormal temperature (hypothermia) and skin changes. However, those clinical signs were related to ROP in univariate analysis.

In BC-group, Cohen’s d indicates a small effect size (D0.18) between D1 and D14 CRP levels. Nevertheless, no significant effect size was reported for CRP in the non-BC group (Table 4). CRP level shows significant drop in the BC group, in comparison to non-BC group with P 0.002(S-Table 7). IVH/PVL and BPD were not significantly different in both study groups, S-Table 2.

#### Adverse outcome and safety

S-Table 7 shows laboratory data in both study groups with no clinically significant difference. No patient in either group had clinically suspected or diagnosed cow milk allergy. In addition, as previously mentioned, feeding intolerance was not significantly different in both study groups.

## Discussion

BC with LDS is an attractive supplement that can help in in promoting growth of preterm infants and prevention of prematurity related complications, in absence of MOM. In this trial, supplementing IF with BC during the first two weeks of life improved the growth parameters and a tendency to reduce ROP incidence in BC group was observed. However, it did not significantly decrease ROP requiring treatment.

### Primary outcome

Few studies searched for effect of BC in premature infants’ first weeks of life on ROP development. Those studies found no significant effect of BC on ROP incidence in premature infants [21–23]. In the current work we found that ROP with its different stages were less common in BC group. However, no significant differences were found in both study groups regarding retinal zones affection, occurrence of Plus /pre-plus disease or ROP requiring anti-VEGF injection.

ROP is a multifactorial disease of the developing retina. No single agent can stop disease pathology and as with most of prematurity-related complications, it requires a bundle of preventive measures. The BC targets multiple causal factors, which can make it suitable for preventing ROP. BC might help in its prevention through possessing anti-inflammatory-antioxidants, antimicrobials, growth-promoting properties, and enhances Hb production.

BC is indeed rich in lactoferrin which has an anti-inflammatory effect. Antioxidants in BC can exert an anti-inflammatory effect through suppression of the expression of pro-inflammatory cytokines [24]. BC has been proven to have anti-inflammatory properties through its ability to decrease CRP levels.

As infection is one of major risk factors for ROP development, especially in LMIC, BC could play a role in its prevention via Ig, lactoperoxidae, lysozymes, and lactoferrin’s antimicrobial properties. Firstly, IgG is the predominant Ig in BC, accounting for 80–90% of all Igs that can compensate for inadequate placental transfer in premature infants [25]. Pasteurization reduces the concentration of Ig and milk bioactive compound in both BC and DM, but a spray-dried BC product may remain highly bioactive for both infants and pigs [26]. Secondly, lactoperoxidase is a potent antimicrobial agent through production of ROS that is harmful to a variety of Gram-positive and Gram-negative bacteria [27]. Thirdly, Gram-negative bacteria are killed by lysozyme’s antibacterial action, which also stops Gram-positive bacteria from growing [28]. Along the hospital stay of studied patients, 7 patients in non-BC group (4 klebsiella and 3 E. coli) had gram negative infection while a lower number was observed in BC group, where four patients had gram negative infection (2 klebsiella and 2 E. coli), S-Table 4. Fourthly, Lactoferrin is the main immunomodulatory component of colostrum of all species. It has antimicrobial properties as it guards against all stages of infection [29]. In the current work, possible sepsis was significantly less in the BC group, and LOS was a significant risk factor for ROP in univariate analysis. In the current work, BC significantly increased weight on D14 and weight-gain. Poor postnatal weight gain and slower postnatal growth velocity have been associated with ROP. Besides the nutritive value of BC, it might be a potentially useful fortifier for the human milk of premature infants [30]. It has higher contents of IGF-1 (500 mg/L in BC compared to 18 mg/L in human colostrum) and more stability of IGF-I during absorption because of its higher contents of casein [31]. IGF-1 promotes development of many tissues, including retinal vessels, and early postnatal low serum IGF-1 is associated with the development of ROP. In addition, BC improved body growth via improving gut functions and immunity, especially in conditions of stress and inflammation, due to its high content of both nutrients (proteins) and immunomodulatory factors [32, 33]. 

In the multivariate analysis, only 3 independent variables were clinically and statistically competent to be in the final model. Only weight gain and birthweight were retained and were significant, therefore, affecting ROP occurrence in addition to BC. BC might have indirectly decreased ROP development through enhancing the weight gain.

Both anemia and blood transfusions can be risk factors for ROP. BC can provide macro-and micro-nutrients (vitamins and minerals) that can enhance erythropoiesis [34]. Moreover, lactoferrin has iron-binding properties, that can enhance intestinal iron absorption and improve hemoglobin production. In the current study, no difference was found between the two groups regarding the development of anemia, and the numbers of blood samples and blood transfusions for each patient. However, despite the fact that the initial hemoglobin levels were similar in the two groups, the hemoglobin level was significantly higher in BC group at the end of study.

### Secondary outcomes and safety

CRP, anemia, PRBCs-transfusion and sepsis were previously discussed as risk factors for ROP, and enhanced growth as a protective factor for ROP in the primary outcome.

BC is a rich source of protein and bioactive components such as lysozyme, lactoperoxidase, Ig, TGF, IGF, and EGF [35]. The relative amino acid composition of BC resembles that of human colostrum. BC may support intestinal adaptation and was recently tested in a pilot study of pediatric patients with short bowel syndrome [36]. In preterm pigs, used as a model for preterm infants, feeding with BC for the first 1–2 weeks improves gut maturation, body growth, and NEC resistance relative to IF and DM [37]. However, in the current work, we found that stage II and III of NEC were similar in both study groups. In addition, feeding intolerance and DTFF were similar in both groups.

BPD and ROP are common and significant morbidities in prematurely born infants. BPD and ROP share the major risk factors of perinatal inflammation and exposure to oxidative stress and have strong genetic predispositions [38]. Both PVL and ROP are related to ischemia and hypoperfusion of the developing retina and brain [39]. In the current work, both PVL and BPD were related to ROP development using univariate regression analysis.

PVL, BPD, NEC, and ROP have common pathogenic pathways. This study proved the potentiality of BC to prevent ROP. Therefore, the ability to prevent other prematurity related complications need more extensive research with higher doses and longer duration. We believe that BC effect on CRP might be a starting point of those future studies.

Osmolarity and allergy should be considered when putting safety in account. There is a high content (70%) of casein in BC that is the main cause of cow milk allergy. However, premature infants are less likely to develop cow’s milk allergy. In the current study, no patient had a clinically-suspected cow milk allergy. High osmolarity can be a risk factor for FI and NEC. Using BC alone, or as a supplement, is unlikely to contribute excessive osmolality since its osmolality is 250–300 mosm/kg, which is lower than the recommended maximum for infant milk diets (400–450 mosm/kg) [37, 40]. 

### Limitations

Lack or insufficiency of MOM is a limitation. Unfortunately, it is the current situations in some NICUs of LMICs. Therefore, IF could be enriched with readily available BC. Long term follow up was not feasible. Human milk can provide protection against prematurity related complications in dose dependent manner [41]. Similarly higher doses of BC for longer duration can be applied, with likely more significant effect on ROP-requiring treatment and other prematurity related complications.

The fact that none of the study’s participants breastfed is a further limitation. Breastfeeding provides inexpensive health advantages for both the mother and the child. Obstetricians and neonatologists have a duty to promote breastfeeding; obstacles should be identified and overcome [42, 43]. On the other hand, BC with LDS may be helpful in situations when MOM is lacking or insufficient, or in unique circumstances such as restricted prenatal or postnatal development, or congenital defects [44–50]. 

Another limitation of the current study is that it is single-centered and a larger sample size might be necessary to assess the impact of BC with LDS on ROP-requiring intervention as it is relatively less prevalent than ROP- not requiring intervention.

## Conclusion

In the absence of MOM and the unavailability of DM, BC with LDS in the first two weeks of life might be helpful in the prevention of those ROP stages which do not require treatment. This might be due to improving growth rate rather than a direct effect. In addition, it enhanced the Hb level on D-14 in premature infants. BC-supplemented IF might be an alternative second choice meal in the absence of MOM, especially in countries with no breast milk banks. However, further multicenter studies with a larger sample size are required to assess the effect of BC with LDS on ROP requiring intervention.

## Electronic supplementary material

Below is the link to the electronic supplementary material.

Supplementary Material 1

## Data Availability

The datasets generated during and/or analyzed during the current study are available from the corresponding author on reasonable request.

## Abbreviations

BC: Bovine colostrum
LDS: Liposomal delivery system
NEC: Necrotizing enterocolitis
BPD: Bronchopulmonary dysplasia
ROP: Retinopathy of prematurity
LOS: Late onset sepsis
PVL: Periventricular leukomalacia
CRP: C-reactive protein
MOM: Mother own milk
IF: Infant formula
DM: Donor milk
Ig: Immunoglobulins
TGF: Transforming growth factors
IGF: Insulin-like growth factors
EGF: Epidermal growth factors ROS reactive oxygen species
LMIC: Low-and-middle income countries
VEGF: Vascular endothelial growth factors
ICROP: International Classification of ROP
FI: Feeding intolerance

## References

[1] Menon G, Williams TC. Human milk for preterm infants: why, what, when and how? Arch Dis Child Fetal Neonatal Ed. 2013;98(6):559–62. 10.1136/archdischild-2012-303582 23893267

[2] Andersson Y, Sävman K, Bläckberg L, Hernell O. Pasteurization of mother’s own milk reduces fat absorption and growth in preterm infants. Acta Paediatr. 2007;96(10):1445–9. 17714541 10.1111/j.1651-2227.2007.00450.x

[3] Schanler RJ, Lau C, Hurst NM, Smith EO. Randomized trial of donor human milk versus preterm formula as substitutes for mothers’ own milk in the feeding of extremely premature infants. Pediatrics. 2005;116(2):400-6. 10.1542/peds.2004-1974. PMID: 16061595. 10.1542/peds.2004-1974 16061595

[4] Corpeleijn WE, de Waard M, Christmann V, van Goudoever JB, Jansen-van der Weide MC, Kooi EM, et al. Effect of donor milk on severe infections and mortality in very low-birth-weight infants: the early nutrition study randomized clinical trial. JAMA Pediatr. 2016;170(7):654–61. 27135598 10.1001/jamapediatrics.2016.0183

[5] Tortadès M, Garcia-Fruitós E, Arís A, Terré M. Short communication: the biological value of transition milk: analyses of immunoglobulin G, IGF-I and lactoferrin in primiparous and multiparous dairy cows. Animal. 2023;41:1008–61. 10.1016/j.animal.2023.100861 37329844

[6] Smith LE. Pathogenesis of retinopathy of prematurity. Semin Neonatol. 2003;8(6):469 – 73. 10.1016/S1084-2756(03)00119-2. PMID: 15001119. 10.1016/S1084-2756(03)00119-2 15001119

[7] Agostoni C, Buonocore G, Carnielli VP, De Curtis M, Darmaun D, Decsi T, et al. Enteral nutrient supply for preterm infants: commentary from the European society of paediatric gastroenterology, hepatology and nutrition committee on nutrition. J Pediatr Gastroenterol Nutr. 2010;50:85e91. 19881390 10.1097/MPG.0b013e3181adaee0

[8] Working group of pediatrics Chinese society of parenteral. And enteral nutrition, working group of neonatology Chinese society of pediatrics, working group of neonatal surgery Chinese society of pediatric surgery. CSPEN guidelines for nutrition support in neonates. Asia Pac J Clin Nutr. 2013;22:655e63. 24231027 10.6133/apjcn.2013.22.4.21

[9] -Nutritional Care of Preterm Infants: Scientific Basis and Practical Guidelines. World Rev Nutr Diet Basel Karger. 2014;110:4–10. 10.1159/000358453).

[10] - Li K, Chen D, Zhao X, et al. Preparation and investigation of Ulex europaeus agglutinin I-conjugated liposomes as potential oral vaccine carriers. Arch Pharm Res. 2011;34:1899–907. 10.1007/s12272-011-1110-3. 22139689 10.1007/s12272-011-1110-3

[11] Gangwe A, Azad RV, Parchand S, Behera S, Re C et al. International Classification of Retinopathy of Prematurity, Third Edition (Ophthalmology. 2021;128:e51-e68). Ophthalmology. 2022;129(3):e36. 10.1016/j.ophtha.2021.10.021. Epub 2021 Nov 26. PMID: 34844764. 10.1016/j.ophtha.2021.10.021 34844764

[12] Chiang MF, Quinn GE, Fielder AR, Ostmo SR, Paul Chan RV, Berrocal A, Binenbaum G, Blair M, Peter Campbell J, Capone A Jr, Chen Y, Dai S, Ells A, Fleck BW, Good WV, Elizabeth Hartnett M, Holmstrom G, Kusaka S, Kychenthal A, Lepore D, Lorenz B, Martinez-Castellanos MA, Özdek Ş, Ademola-Popoola D, Reynolds JD, Shah PK, Shapiro M, Stahl A, Toth C, Vinekar A, Visser L, Wallace DK, Wu WC, Zhao P, Zin A. International classification of retinopathy of Prematurity, Third Edition. Ophthalmology. 2021;128(10):e51–68. 10.1016/j.ophtha.2021.05.031. Epub 2021 Jul 8. PMID: 34247850. 34247850 10.1016/j.ophtha.2021.05.031 PMC10979521

[13] Walsh MC, Kliegman RM. Necrotizing enterocolitis: treatment based on staging criteria. Pediatr Clin North Am. 1986;33:179e201. 3081865 10.1016/S0031-3955(16)34975-6 PMC7131118

[14] Kasat K, Hendricks-Muñoz KD, Mally PV. Neonatal red blood cell transfusions: searching for better guidelines. Blood Transfus. 2011;9(1):86–94. 10.2450/2010.0031-10. PMID: 21235854; PMCID: PMC3021402. 21235854 10.2450/2010.0031-10 PMC3021402

[15] Haque KN. Definitions of bloodstream infection in the newborn. Pediatr Crit Care Med. 2005;6(3 Suppl):S45-9. 10.1097/01.PCC.0000161946.73305.0A. PMID: 15857558. 10.1097/01.PCC.0000161946.73305.0A 15857558

[16] Sun L, Zhang H, Bao Y, Li W, Wu J, He Y, Zhu J. Long-term outcomes of Bronchopulmonary Dysplasia under two different diagnostic criteria: a retrospective cohort study at a Chinese Tertiary Center. Front Pediatr. 2021;9:648972. 10.3389/fped.2021.648972. PMID: 33859971; PMCID: PMC8042161. 33859971 10.3389/fped.2021.648972 PMC8042161

[17] Burris AD, Burris J, Järvinen KM. Cow’s milk protein allergy in term and Preterm infants: clinical manifestations, immunologic pathophysiology, and management strategies. Neoreviews. 2020;21(12):e795–808. 10.1542/neo.21-12-e795. PMID: 33262206. 33262206 10.1542/neo.21-12-e795

[18] Ochoa TJ, Zegarra J, Bellomo S, Carcamo CP, Cam L, Castañeda A, Villavicencio A, Gonzales J, Rueda MS, Turin CG, Zea-Vera A, Guillen D, Campos M, Ewing-Cobbs L, NEOLACTO Research Group. Randomized controlled trial of bovine lactoferrin for Prevention of Sepsis and Neurodevelopment Impairment in infants weighing Less Than 2000 Grams. J Pediatr. 2020;219:118–e1255. 10.1016/j.jpeds.2019.12.038. 32037149 10.1016/j.jpeds.2019.12.038 PMC7096260

[19] Ahnfeldt AM, Aunsholt L, Hansen BM, Hoest B, Jóhannsdóttir V, Kappel SS, Klamer A, Möller S, Moeller BK, Sangild PT, Skovgaard AL, van Hall G, Vibede LD, Zachariassen G. Bovine colostrum as a fortifier to human milk in very preterm infants - a randomized controlled trial (FortiColos). Clin Nutr. 2023;42(5):773–83. 10.1016/j.clnu.2023.03.008. 37004355 10.1016/j.clnu.2023.03.008

[20] Muralidharan K. On sample size determination. Math J Interdisciplinary Sci. 2014;3(1):55–64. 10.15415/mjis.2014.31005.

[21] Juhl SM, Ye X, Zhou P, Li Y, Iyore EO, Zhang L, Jiang P, van Goudoever JB, Greisen G, Sangild PT. Bovine Colostrm for Preterm Infants in the First Days of Life: A Randomized Controlled Pilot Trial. J Pediatr Gastroenterol Nutr. 2018;66(3):471–478. 10.1097/MPG.0000000000001774. PMID: 29019855. 10.1097/MPG.0000000000001774 29019855

[22] Yan X, Pan X, Ding L, Dai Y, Chen J, Yang Y, Li Y, Hao H, Qiu H, Ye Z, Shen RL, Li Y, Ritz C, Peng Y, Zhou P, Gao F, Jiang PP, Lin HC, Zachariassen G, Sangild PT, Wu B. Bovine colostrum to supplement the first feeding of very preterm infants: the PreColos randomized controlled trial. Clin Nutr. 2023;42(8):1408–17. Epub 2023 Jun 28. PMID: 37437359. 37437359 10.1016/j.clnu.2023.06.024

[23] Ahnfeldt AM, Aunsholt L, Hansen BM, Hoest B, Jóhannsdóttir V, Kappel SS, et al. Bovine colostrum as a fortifier to human milk in very preterm infants - a randomized controlled trial (FortiColos). Clin Nutr. 2023;42(5):773–83. 37004355 10.1016/j.clnu.2023.03.008

[24] Mucha P, Skoczyńska A, Małecka M, Hikisz P, Budzisz E. Overview of the antioxidant and anti-inflammatory activities of selected plant compounds and their metal ions complexes. Molecules. 2021;26(16):4886. 10.3390/molecules26164886. PMID: 34443474; PMCID: PMC8398118. 34443474 10.3390/molecules26164886 PMC8398118

[25] Stelwagen K, Carpenter E, Haigh B, Hodgkinson A, Wheeler TT. Immune components of bovine colostrum and milk. J Anim Sci. 2009;87:3–9. 18952725 10.2527/jas.2008-1377

[26] Stoy ACF, Sangild PT, Skovgaard K, Thymann T, Bjerre M, Chatterton DEW, et al. Spray dried, pasteurised bovine colostrum protects against gut dysfunction and inflammation in preterm pigs. J Pediatr Gastroenterol Nutr. 2016;63(2):280–7. 26756878 10.1097/MPG.0000000000001056

[27] Seifu E, Buys EM, Donkin EF. Significance of the lactoperoxidase system in the dairy industry and its potential applications: a review. Trends Food Sci Technol. 2005;16:137–45.

[28] Wheeler TT, Hodgkinson AJ, Prosser CG, Davis SR. Immune components of colostrum and milk—A historical perspective. J Mammary Gland Biol Neoplasia. 2007;12:237–47. 17992474 10.1007/s10911-007-9051-7

[29] Farag M, Badr-Eldin O, Attia M, Morsi N, El-haddad. R.Effect of lactoferrin in the prevention of late-onset sepsis in preterm neonates: a randomized-controlled trial. Alexandria Journal of Pediatrics 34(1):p 1–9, Jan–Apr 2021. | 10.4103/ajop.ajop_11_21

[30] Poulsen AR, de Jonge N, Sugiharto S, Nielsen JL, Lauridsen C, Canibe N. The microbial community of the gut differs between piglets fed sow milk, milk replacer or bovine colostrum. Br J Nutr. 2017;117(7):964–78. 28460652 10.1017/S0007114517000216

[31] Kimura T, Murakawa Y, Ohno M, Ohtani S, Higaki K. Gastrointestinal absorption of recombinant human insulin-like growth factor-I in rats. J Pharmacol Exp Ther. 1997;283:611–8. 9353376

[32] Cekmez F, Pirgon O, Aydemir G, Dundar B, Cekmez Y, Karaoglu A, Fidanc K, Tunc T, Aydinoz S, Karademir F, Süleymanoglu S. Correlation between cord blood apelin and IGF-1 levels in retinopathy of prematurity. Biomark Med. 2012;6(6):821–5. 23227848 10.2217/bmm.12.82

[33] Pérez-Muñuzuri A, Fernández-Lorenzo JR, Couce-Pico ML, BlancoTeijeiro MJ, Fraga-Bermúdez JM. Serum levels of IGF1 are a useful predictor of retinopathy of prematurity. Acta Paediatr. 2010;99(4):519–25. 20085549 10.1111/j.1651-2227.2009.01677.x

[34] Playford RJ, Weiser MJ. Bovine colostrum: its constituents and uses. Nutrients. 2021;13:265. 33477653 10.3390/nu13010265 PMC7831509

[35] Chatterton DE, Nguyen DN, Bering SB, Sangild PT. Anti-inflammatory mechanisms of bioactive milk proteins in the intestine of newborns. Int J Biochem Cell Biol. 2013;45(8):1730–47. 23660296 10.1016/j.biocel.2013.04.028

[36] Aunsholt L, Qvist N, Sangild PT, Vegge A, Stoll B, Burrin DG, et al. Minimal enteral nutrition to improve adaptation after intestinal resection in piglets and infants. J Parenter Enter Nutr. 2018;42(2):446–54. 10.1177/0148607117690527 28786308

[37] Shen RL, Thymann T, Østergaard MV, Støy AC, Krych Ł, Nielsen DS, et al. Early gradual feeding with bovine colostrum improves gut function and NEC resistance relative to infant formula in preterm pigs. Am J Physiol Gastrointest Liver Physiol. 2015;309(5):310–23. 10.1152/ajpgi.00163.2015 26138468

[38] Stark A, Dammann C, Nielsen HC, Volpe MV. A pathogenic relationship of Bronchopulmonary Dysplasia and Retinopathy of Prematurity? A review of angiogenic mediators in both diseases. Front Pediatr. 2018;6:125. 10.3389/fped.2018.00125. PMID: 29951473; PMCID: PMC6008318. 29951473 10.3389/fped.2018.00125 PMC6008318

[39] Huang HM, Lin SA, Chang YC, Kuo HK. Correlation between periventricular leukomalacia and retinopathy of prematurity. Eur J Ophthalmol. 2012 Nov-Dec;22(6):980–4. 10.5301/ejo.5000129. Epub 2012 Feb 27. PMID: 22388777. 10.5301/ejo.5000129 22388777

[40] Choi A, Fusch G, Rochow N, Fusch C. Target fortification of breast milk: Predicting the final osmolality of the feeds. PLoS ONE. 2016;11(2):1–12. 10.1371/journal.pone.0148941 PMC4749227 26863130

[41] Xu Y, Yu Z, Li Q, Zhou J, Yin X, Ma Y, Yin Y, Jiang S, Zhu R, Wu Y, Han L, Gao Y, Xue M, Qiao Y, Zhu L, Tu W, Wu M, Wan J, Wang W, Deng X, Li S, Wang S, Chen X, Zhou Q, Wang J, Cheng R, Wang J, Han S. Dose-dependent effect of human milk on bronchopulmonary dysplasia in very low birth weight infants. BMC Pediatr. 2020;20(1):522. 10.1186/s12887-020-02394-1. PMID: 33190629; PMCID: PMC7666971. 33190629 10.1186/s12887-020-02394-1 PMC7666971

[42] The social role of pediatrics in the past and present times, Serra G, Giuffrè M, Piro E, Corsello G. Ital J Pediatr. 2021;47:239. 34922600 10.1186/s13052-021-01190-6 PMC8684095

[43] Serra G, Miceli V, Albano S, Corsello G. Perinatal and newborn care in a two years retrospective study in a first level peripheral hospital in Sicily (Italy). Ital J Pediatr. 2019;45:152. 31783883 10.1186/s13052-019-0751-6 PMC6884854

[44] Piro E, Serra G, Schierz IAM, Giuffrè M, Corsello G. Fetal growth restriction: a growth pattern with fetal, neonatal and long-term consequences. Euromediterranean Biomedical J. 2019;14(09):038–44.

[45] Schierz IAM, Serra G, Antona V, Persico I, Corsello G, Piro E. Infant developmental profile of Crisponi syndrome due to compound heterozygosity for CRLF1 deletion. Clin Dysmorphol. 2020;29(3):141–143. 10.1097/MCD.0000000000000325. PMID: 32433043. 10.1097/MCD.0000000000000325 32433043

[46] 2q13 microdeletion. Syndrome: report on a newborn with additional features expanding the phenotype. Ettore Piro, Gregorio Serra, Mario Giuffrè, Ingrid Anne Mandy Schierz, Giovanni Corsello. Clin Case Rep. 2021;9:1–5. 10.1002/ccr3.4289.

[47] Piro E, Serra G, Schierz IAM, Giuffrè M, Corsello G. Neonatal ten-year retrospective study on neural tube defects in a second level University Hospital. Ital J Pediatr. 2020;46(1):72. 10.1186/s13052-020-00836-1. PMID: 32448340; PMCID: PMC7247239. 32448340 10.1186/s13052-020-00836-1 PMC7247239

[48] Serra G, Antona V, Giuffrè M, Piro E, Salerno S, Schierz IAM, Corsello G. Interstitial deletions of chromosome 1p: novel 1p31.3p22.2 microdeletion in a newborn with craniosynostosis, coloboma and cleft palate, and review of the genomic and phenotypic profiles. Ital J Pediatr. 2022;48(1):38. 10.1186/s13052-022-01232-7. PMID: 35246213; PMCID: PMC8896361. 35246213 10.1186/s13052-022-01232-7 PMC8896361

[49] Serra G, Giambrone C, Antona V, Cardella F, Carta M, Cimador M, Corsello G, Giuffrè M, Insinga V, Maggio MC, Pensabene M, Schierz IAM, Piro E. Congenital hypopituitarism and multiple midline defects in a newborn with non-familial Cat Eye syndrome. Ital J Pediatr., Serra G, Memo L, Cavicchioli P, Cutrone M, Giuffrè M, La Torre ML, Schierz IAM, Corsello G. Novel mutations of the ABCA12, KRT1 and ST14 genes in three unrelated newborns showing congenital ichthyosis. Ital J Pediatr. 2022;48(1):145.

[50] Schierz IAM, Giuffrè M, Cimador M, D’Alessandro MM, Serra G, Favata F, Antona V, Piro E, Corsello G. Hypertrophic pyloric stenosis masked by kidney failure in a male infant with a contiguous gene deletion syndrome at Xp22.31 involving the steroid sulfatase gene: case report. Ital J Pediatr. 2022;48:19. 35115028 10.1186/s13052-022-01218-5 PMC8812169

