# Supplementary material for: Do preterm infants’ retinas like bovine colostrum? A randomized controlled trial
Source: Ital J Pediatr. 2024 Oct 19;50:218. doi: 10.1186/s13052-024-01781-z (PMC11490996; doi:10.1186/s13052-024-01781-z)

**S-Table (1): Comparison between the two studied groups as regards anthropometric measurements**

|  | **Groups** | | **Test of sig.** | **P** |
| --- | --- | --- | --- | --- |
|  | **B. Colostrum** | **Control** |  |  |
| **WT at admission (gms)** |  |  |  |  |
| Min. – Max. | 760.0 – 1670.0 | 670.0 – 1800.0 | t= -1.738 | 0.084 |
| Mean ± SD. | 1179.1 ± 208.6 | 1231.2 ± 215.6 |  |  |
| Median (IQR) | 1175.0 (1000.0 – 1350.0) | 1230.0 (1065.0 – 1400.0) |  |  |
| **WT at 14th day (gms)** |  |  |  |  |
| Min. – Max. | 825.0 – 1725.0 | 660.0 – 1840.0 | t= 1.847 | 0.066 |
| Mean ± SD. | 1297.0 ± 207.7 | 1242.1 ± 215.8 |  |  |
| Median (IQR) | 1308.5 (1132.5 – 1465.0) | 1247.5 (1092.5 – 1395.0) |  |  |
| **WT gain in first 14 days (grams)** |  |  |  |  |
| Min. – Max. | 2.0 – 260.0 | -250.0 – 140.0 | U= 789.5 | <0.001**^*^** |
| Mean ± SD. | 118.3 ± 52.7 | 10.9 ± 61.6 |  |  |
| Median (IQR) | 119.0 (85.0 – 149.0) | 25.0 (-20.0 – 51.5) |  |  |
| **WT at time of ROP screen (gm )** |  |  |  |  |
| Min. – Max. | 1005.0 – 1900.0 | 710.0 – 1950.0 | U= 4244.5 | 0.065 |
| Mean ± SD. | 1437.0 ± 201.1 | 1373.9 ± 225.6 |  |  |
| Median (IQR) | 1460.0 (1257.5 – 1600.0) | 1386.0 (1200.0 – 1555.0) |  |  |
| **WT at discharge (gm )** | **(n = 95)** | **(n = 92)** |  |  |
| Min. – Max. | 1500.0 – 1780.0 | 1500.0 – 1840.0 | U= 3739 | 0.086 |
| Mean ± SD. | 1556.4 ± 62.9 | 1538.9 ± 49.6 |  |  |
| Median (IQR) | 1530.0 (1510.0 – 1600.0) | 1520.0 (1510.0 – 1560.0) |  |  |
| **HC at admission(cm)** |  |  |  |  |
| Min. – Max. | 22.0 – 32.0 | 23.0 – 31.5 | U= 4712 | 0.477 |
| Mean ± SD. | 27.4 ± 1.9 | 27.5 ± 1.9 |  |  |
| Median (IQR) | 27.0 (26.0 – 29.0) | 28.0 (26.0 – 29.0) |  |  |
| **HC 1st week (cm)** |  |  |  |  |
| Min. – Max. | 23.0 – 32.0 | 23.5 – 32.0 | U= 4705 | 0.469 |
| Mean ± SD. | 27.7 ± 1.9 | 27.8 ± 1.9 |  |  |
| Median (IQR) | 27.8 (26.0 – 29.0) | 28.0 (26.4 – 29.0) |  |  |
| **HC 2nd week (cm)** |  |  |  |  |
| Min. – Max. | 23.0 – 32.5 | 24.0 – 32.5 | U= 4927 | 0.858 |
| Mean ± SD. | 28.4 ± 1.8 | 28.4 ± 1.9 |  |  |
| Median (IQR) | 28.2 (27.0 – 30.0) | 28.5 (27.0 – 30.0) |  |  |
| **HC at discharge (Cm)** | **(n = 95)** | **(n = 92)** |  |  |
| Min. – Max. | 27.5 – 33.0 | 28.5 – 34.5 | U= 4138 | 0.528 |
| Mean ± SD. | 30.8 ± 1.1 | 30.9 ± 1.2 |  |  |
| Median (IQR) | 31.0 (30.0 – 31.5) | 31.0 (30.0 – 32.0) |  |  |
| **Length at admission (days)** |  |  |  |  |
| Min. – Max. | 31.0 – 46.0 | 32.0 – 41.0 | U= 4415 | 0.148 |
| Mean ± SD. | 37.8 ± 2.2 | 37.3 ± 1.9 |  |  |
| Median (IQR) | 38.0 (36.0 – 39.0) | 38.0 (36.0 – 39.0) |  |  |
| **Length at discharge (cm)** | **(n = 95)** | **(n = 92)** |  |  |
| Min. – Max. | 35.0 – 48.0 | 38.0 – 43.0 | U= 3891 | 0.189 |
| Mean ± SD. | 40.6 ± 1.5 | 40.4 ± 1.2 |  |  |
| Median (IQR) | 40.7 (40.0 – 41.0) | 40.0 (39.5 – 41.0) |  |  |

U: Mann Whitney test p: p value for comparing between the studied groups *: Statistically significant at p ≤ 0.05

HC: Head circumference WT: Weight t: Student t-test

**S-Table (2): Comparison between the two studied groups as regards in-hospital course, prematurity related complications and mortality.**

|  | | **Groups** | | | | **Test of sig.** | **P** |
| --- | --- | --- | --- | --- | --- | --- | --- |
|  |  | **B. Colostrum** | | **Control** | |  |  |
| **Apnea** | No | 41 (41.0%) | | 38 (38.0%) | | χ^2^= 0.188 | 0.664 |
|  | Yes | 59 (59.0%) | | 62 (62.0%) | |  |  |
| **Feeding intolerance** | No | 58 (58.0%) | | 52 (52.0%) | | χ^2^= 0.727 | 0.394 |
|  | Yes | 42 (42.0%) | | 48 (48.0%) | |  |  |
| **Tachycardia** | No | 62 (62.0%) | | 50 (50.0%) | | χ^2^= 2.922 | 0.087 |
|  | Yes | 38 (38.0%) | | 50 (50.0%) | |  |  |
| **Hypoactivity** | No | 70 (70.0%) | | 43 (43.0%) | | χ^2^= 14.831 | <0.001**^*^** |
|  | Yes | 30 (30.0%) | | 57 (57.0%) | |  |  |
| **NEC StageII/III** | No | 100 (100.0%) | | 98 (98.0%) | | χ^2^= 2.020 | FEp= 0.497 |
|  | Yes | 0 (0.0%) | | 2 (2.0%) | |  |  |
| **BPD** | No | 100 (100.0%) | | 95 (95.0%) | | χ^2^= 5.128 | FEp= 0.059 |
|  | Yes | 0 (0.0%) | | 5 (5.0%) | |  |  |
| **IVH/PVL** | No | 39 (39.0%) | | 34 (34.0%) | | χ^2^= 0.539 | 0.463 |
|  | Yes | 61 (61.0%) | | 66 (66.0%) | |  |  |
| **Hypothermia attacks** | No | 84 (84.0%) | | 87 (87.0%) | | χ^2^= 0.363 | 0.547 |
|  | Yes | 16 (16.0%) | | 13 (13.0%) | |  |  |
| **Skin changes** | No | 97 (97.0%) | | 91 (91.0%) | | χ^2^= 3.191 | 0.074 |
|  | Yes | 3 (3.0%) | | 9 (9.0%) | |  |  |
| **Oxygen/CPAP** | No | 26 (26.0%) | | 13 (13.0%) | | χ^2^= 5.383 | 0.020**^*^** |
|  | Yes | 74 (74.0%) | | 87 (87.0%) | |  |  |
| **Invasive Ventilation** | No | 77 (77.0%) | | 79 (79.0%) | | χ^2^= 0.117 | 0.733 |
|  | Yes | 23 (23.0%) | | 21 (21.0%) | |  |  |
| **Ventilation duration** | | **(n = 23)** | | **(n = 21)** | |  |  |
| Min. – Max. | | 1.0 – 6.0 | | 1.0 – 5.0 | | U= 216 | 0.539 |
| Mean ± SD. | | 3.3 ± 1.6 | | 3.0 ± 1.5 | |  |  |
| Median (IQR) | | 3.0 (2.0 – 5.0) | | 3.0 (2.0 – 4.0) | |  |  |
| **Inotropes** | No | 94 (94.0%) | | 93 (93.0%) | | χ^2^= 0.082 | 0.774 |
|  | Yes | 6 (6.0%) | | 7 (7.0%) | |  |  |
| **Days to reach full feed** | | |  | |  |  |  |
| Min. – Max. | | | 5.0 – 18.0 | | 5.0 – 18.0 | U= 4589 | 0.313 |
| Mean ± SD. | | | 10.3 ± 3.0 | | 10.7 ± 3.3 |  |  |
| Median (IQR) | | | 10.0 (8.0 – 12.0) | | 11.0 (8.0 – 13.0) |  |  |
| **Hospital stay duration** | | |  | |  |  |  |
| Min. – Max. | | | 14.0 – 86.0 | | 14.0 – 90.0 | U= 4735 | 0.517 |
| Mean ± SD. | | | 40.6 ± 18.1 | | 42.7 ± 20.0 |  |  |
| Median (IQR) | | | 36.0 (27.0 – 51.5) | | 40.0 (28.0 – 58.0) |  |  |
| **Fate** | Discharged | | 95 (95.0%) | | 91 (91.0%) | χ^2^= 1.229 | 0.268 |
|  | Died | | 5 (5.0%) | | 9 (9.0%) |  |  |

**χ^2^**: Chi square test U: Mann Whitney test **FE: Fisher Exact**

p: p value for comparing between the studied groups *: Statistically significant at p ≤ 0.05

EOS: Early onset sepsis LOS: Late onset sepsis NEC: Necrotizing enterocolitis

BPD: Bronchopulmonary dysplasia IVH: Intraventricular hemorrhage

PVL: Periventricular leukomalacia

**S-Table (3): Comparison between the two studied groups as regards anemia, Erythropoietin intake, HB Day 1and 14, blood samples and blood transfusion.**

|  | | | **Groups** | | **Test of sig.** | **P** |
| --- | --- | --- | --- | --- | --- | --- |
|  |  |  | **B. Colostrum** | **Control** |  |  |
| **Anemia** | | NO | 11 (11.0%) | 14 (14.0%) | χ^2^= 0.411 | 0.521 |
|  |  | Yes | 89 (89.0%) | 86 (86.0%) |  |  |
| **Erythropoietin** | No | | 38 (38%)  62 (62%) | 36 (36%)  64 (64%) | 1.086 | 0.770 |
|  | Yes | |  |  |  |  |
| **HB-D1 (g/dl)** | | |  |  |  |  |
| Min. – Max. | | | 9.8 – 21.6 | 9.1 – 23.5 | t= -1.259 | 0.209 |
| Mean ± SD. | | | 15.6 ± 2.2 | 16.0 ± 2.3 |  |  |
| Median (IQR) | | | 15.7 (14.4 – 16.6) | 16.2 (14.6 – 17.5) |  |  |
| **HB-D14 (g/dl)** | | |  |  |  |  |
| Min. – Max. | | | 8.7 – 16.8 | 8.0 – 19.5 | U= 4056.5 | 0.021**^*^** |
| Mean ± SD. | | | 12.4 ± 1.8 | 11.8 ± 2.0 |  |  |
| Median (IQR) | | | 12.2 (11.0 – 13.9) | 11.7 (10.5 – 12.9) |  |  |
| **Blood samples** | | |  |  |  |  |
| Min. – Max. | | | 5.0 – 23.0 | 4.0 – 22.0 | U= 4377 | 0.127 |
| Mean ± SD. | | | 11.5 ± 4.2 | 12.4 ± 4.6 |  |  |
| Median (IQR) | | | 11.0 (8.0 – 14.0) | 12.5 (8.0 – 16.0) |  |  |
| **Blood transfusion** | | No | 39 (39.0%) | 38 (38.0%) | χ ^2^= 0.021 | 0.884 |
|  |  | Yes | 61 (61.0%) | 62 (62.0%) |  |  |
| **Times received blood transfusion** | | |  |  |  |  |
| Min. – Max. | | | 1.0 – 5.0 | 1.0 – 6.0 | U= 1615.5 | 0.148 |
| Mean ± SD. | | | 2.1 ± 1.2 | 2.4 ± 1.2 |  |  |
| Median (IQR) | | | 2.0 (1.0 – 3.0) | 2.0 (2.0 – 3.0) |  |  |

**χ^2^**: Chi square test U: Mann Whitney test

p: p value for comparing between the studied groups *: Statistically significant at p ≤ 0.05

**S-Table (4): Comparison between the two studied groups as regards sepsis occurrence according to EMA criteria (2011)**

|  | | **Groups** | | **Test of sig.** | **P** |
| --- | --- | --- | --- | --- | --- |
|  |  | **B. Colostrum** | **Non-BC** |  |  |
| **EOS#** | No | 77 (77.0%) | 60 (60.0%) | χ^2^= 6.697 | 0.010**^*^** |
|  | Yes | 23 (23.0%) | 40 (40.0%) |  |  |
| **LOS** | No | 70 (70.0%) | 67 (67.0%) | χ^2^= 0.209 | 0.648 |
|  | Yes | 30 (30.0%) | 33 (33.0%) |  |  |
| **Proved sepsis** | |  |  |  |  |
| **BC at admission** | Sterile | 96 (96.0%) | 95 (95.0%) | χ^2^= 0.116 | FEp= 1.000 |
|  | Positive | 4 (4.0%)1 | 5 (5.0%)2 |  |  |
| **BC (between 14-28)** | Sterile | 47 (94.0%) | 43 (89.6%) | χ^2^= 0.637 | FEp= 0.482 |
|  | Positive | 3 (6.0%)3 | 5 (10.4%)4 |  |  |
| **BC**  **(before discharge)** | Sterile | 3 (100.0%) | 5 (100.0%) | − | − |
|  | Positive | 0 (0%) | 0 (0%) |  |  |
| **Probable sepsis** | No | 67 (67.0%) | 65 (65.0%) | χ^2^= 0.089 | 0.765 |
|  | Yes | 33 (33.0%) | 35 (35.0%) |  |  |
| **Suspected sepsis** | No | 51 (51.0%) | 31 (31.0%) | χ^2^= 8.268 | 0.004**^*^** |
|  | Yes | 49 (49.0%) | 69 (69.0%) |  |  |

**χ^2^**: Chi square test FE: Fisher Exact p: p value for comparing between the studied groups

*: Statistically significant at p ≤ 0.05

BC: Blood culture EOS early onset sepsis, LOS late onset sepsis

The second BC was done D3-D14

Follow up BC was done after 14 days

(1)one klebsiella, one enterococci, two E-coli

(2)One klebsiella, One streptococci, Three E-coli

(3)One klebsiella, Two candida

(4)One MRSA, One streptococci, One enterococci, Three klebsiella

**S-Table (5): Comparison between the two studied groups as regards retinopathy of prematurity (stages ,zones and presence of Pre/plus disease).**

|  | | **Groups** | | **Test of sig.** | **P** |
| --- | --- | --- | --- | --- | --- |
|  |  | **B. Colostrum** | **Non-BC** |  |  |
| **Stage** | **0** | **95^a^ (95.0%)** | **84^b^ (84.0%)** | χ^2^=0**7.076** | **^MC^p = 0.041^*^** |
|  | **1** | **3^a^ (4.0%)** | **12^b^ (13.0%)** |  |  |
|  | **2** | **1^a^ (1.0%)** | **1^a^ (3.0%)** |  |  |
|  | **3** | **1^a^ (1.0%)** | **3^a^ (3.0%)** |  |  |
| **Zone** | **II** | **2 (2.0%)** | **6 (6.0%)** | χ^2^=0**2.083** | **FEp= 0.279** |
|  | **III** | **98 (98.0%)** | **94 (94.0%)** |  |  |
| **Plus** | **No** | **99 (99.0%)** | **99 (99.0%)** | χ^2^=0**0** | **1.000** |
|  | **Pre plus** | **1 (1.0%)** | **1 (1.0%)** |  |  |
|  | **Plus** | **0 (0.0%)** | **0 (0.0%)** |  |  |

**χ^2^**: Chi square test **MC**: Monte Carlo FE: Fisher Exact

p: p value for comparing between the studied groups

*: Statistically significant at p ≤ 0.05

**In each row: different letters are significant**

**S-Table (6a):Univariate analysis for ROP (presence and absence) as regards demographic, delivery and resuscitation data**

|  | | **ROP** | | **OR  (95% C.I)** | **P** |
| --- | --- | --- | --- | --- | --- |
|  |  | **No** | **Yes** |  |  |
| **GA** | |  |  |  |  |
| Min. – Max. | | 27.0 – 32.0 | 27.0 – 32.0 | 0.534 (0.373 – 0.765) | <0.001**^*^** |
| Mean ± SD. | | 30.6 ± 1.2 | 29.5 ± 1.5 |  |  |
| Median (IQR) | | 31.0 (30.0 – 32.0) | 30.0 (29.0 – 30.0) |  |  |
| **BWT** | |  |  |  |  |
| Min. – Max. | | 790.0 – 1800.0 | 670.0 – 1380.0 | 0.995 (0.992 – 0.998) | <0.001**^*^** |
| Mean ± SD. | | 1225.1 ± 208.8 | 1034.8 ± 174.1 |  |  |
| Median (IQR) | | 1230 (1040 – 1390) | 1030 (920 – 1100) |  |  |
| **WT gain** | |  |  |  |  |
| Min. – Max. | | -250.0 – 260.0 | -130.0 – 140.0 | 0.991 (0.985 – 0.997) | 0.003**^*^** |
| Mean ± SD. | | 70.5 ± 76.9 | 14.1 ± 75.7 |  |  |
| Median (IQR) | | 70.0 (28.0 – 120.0) | 25.0 (-20.0 – 60.0) |  |  |
| **Sex** | Male | 98 (54.7%) | 10 (47.6%) | 0.751 (0.304 – 1.858) | 0.536 |
|  | Female | 81 (45.3%) | 11 (52.4%) | 1.000 |  |
| **MOD** | CS | 139 (77.7%) | 13 (61.9%) | 0.468 (0.181 – 1.207) | 0.116 |
|  | NVD | 40 (22.3%) | 8 (38.1%) | 1.000 |  |
| **Antenatal steroids** | Complete | 63 (35.2%) | 5 (23.8%) | 0.476 (0.147 – 1.548) | 0.217 |
|  | Incomplete | 68 (38.0%) | 8 (38.1%) | 0.706 (0.248 – 2.012) | 0.515 |
|  | No | 48 (26.8%) | 8 (38.1%) | 1.000 |  |
| **Resuscitation** | Initial steps | 134 (74.9%) | 16 (76.2%) | 1.000 |  |
|  | PPV (Ambu) | 39 (21.8%) | 4 (19.0%) | 0.859 (0.271 – 2.719) | 0.796 |
|  | PPV (ETT) | 6 (3.4%) | 1 (4.8%) | 1.396 (0.158 – 12.342) | 0.764 |
| **APGAR at 1 min** | |  |  |  |  |
| Min. – Max. | | 3.0 – 8.0 | 3.0 – 8.0 | 1.012 (0.709 – 1.445) | 0.947 |
| Mean ± SD. | | 6.0 ± 1.3 | 6.0 ± 1.4 |  |  |
| Median (IQR) | | 6.0 (6.0 – 7.0) | 7.0 (6.0 – 7.0) |  |  |
| **APGAR at 5 min** | |  |  |  |  |
| Min. – Max. | | 5.0 – 9.0 | 5.0 – 9.0 | 1.089 (0.700 – 1.695) | 0.705 |
| Mean ± SD. | | 7.7 ± 1.0 | 7.8 ± 1.2 |  |  |
| Median (IQR) | | 8.0 (7.0 – 8.0) | 8.0 (8.0 – 9.0) |  |  |

OR: **Odd`s ratio** C.I: Confidence interval

p: p value for **Odd`s ratio** for comparing between the studied groups.

**S-Table (6b): Univariate analysis for ROP (presence and absence) as regards associated morbidities and complications**

|  | | **ROP** | | **OR (95% C.I)** | **P** |
| --- | --- | --- | --- | --- | --- |
|  |  | **No** | **Yes** |  |  |
| **EOS** | No | 125 (69.8%) | 12 (57.1%) | 1.000 |  |
|  | Yes | 54 (30.2%) | 9 (42.9%) | 1.736 (0.691 – 4.362) | 0.241 |
| **LOS** | No | 130 (72.6%) | 7 (33.3%) | 1.000 |  |
|  | Yes | 49 (27.4%) | 14 (66.7%) | 5.306 (2.022 – 13.927) | <0.001**^*^** |
| **Apnea** | No | 76 (42.5%) | 3 (14.3%) | 1.000 |  |
|  | Yes | 103 (57.5%) | 18 (85.7%) | 4.427 (1.259 – 15.571) | 0.020**^*^** |
| **FI** | No | 104 (58.1%) | 6 (28.6%) | 1.000 |  |
|  | Yes | 75 (41.9%) | 15 (71.4%) | 3.467 (1.285 – 9.350) | 0.014**^*^** |
| **Tachycardia** | No | 106 (59.2%) | 6 (28.6%) | 1.000 |  |
|  | Yes | 73 (40.8%) | 15 (71.4%) | 3.630 (1.345 – 9.795) | 0.011**^*^** |
| **Hypoactivity** | No | 107 (59.8%) | 6 (28.6%) | 1.000 |  |
|  | Yes | 72 (40.2%) | 15 (71.4%) | 3.715 (1.377 – 10.026) | 0.010**^*^** |
| **NEC** | No | 177 (98.9%) | 21 (100.0%) | 1.000 |  |
|  | Yes | 2 (1.1%) | 0 (0.0%) | – | 0.999 |
| **BPD** | No | 177 (98.9%) | 18 (85.7%) | 1.000 |  |
|  | Yes | 2 (1.1%) | 3 (14.3%) | 14.750 (2.311 – 94.158) | 0.004**^*^** |
| **IVH/PVL** | No | 70 (39.1%) | 3 (14.3%) | 1.000 |  |
|  | Yes | 109 (60.9%) | 18 (85.7%) | 3.853 (1.095 – 13.565) | 0.036**^*^** |
| **Thermal abnormality (hypothermia)** | No | 157 (87.7%) | 14 (66.7%) | 1.000 |  |
|  | Yes | 22 (12.3%) | 7 (33.3%) | 3.568 (1.298 – 9.807) | 0.014**^*^** |
| **Anemia** | No | 23 (12.8%) | 2 (9.5%) | 1.000 |  |
|  | Yes | 156 (87.2%) | 19 (90.5%) | 1.401 (0.306 – 6.413) | 0.664 |
| **Oxygen/CPAP** | No | 38 (21.2%) | 1 (4.8%) | 1.000 |  |
|  | Yes | 141 (78.8%) | 20 (95.2%) | 5.390 (0.701 – 41.456) | 0.106 |
| **Invasive Ventilation** | No | 145 (81.0%) | 11 (52.4%) | 1.000 |  |
|  | Yes | 34 (19.0%) | 10 (47.6%) | 3.877 (1.523 – 9.868) | 0.004**^*^** |
| **Ventilation duration** | |  |  |  |  |
| Min. – Max. | | 1.0 – 6.0 | 1.0 – 6.0 | 1.181 (0.739 – 1.887) | 0.486 |
| Mean ± SD. | | 3.1 ± 1.5 | 3.5 ± 1.6 |  |  |
| Median (IQR) | | 3.0 (2.0 – 5.0) | 3.5 (2.0 – 5.0) |  |  |
| **Inotropes** | No | 169 (94.4%) | 18 (85.7%) | 1.000 |  |
|  | Yes | 10 (5.6%) | 3 (14.3%) | 2.817 (0.710 – 11.181) | 0.141 |

OR: **Odd`s ratio** C.I: Confidence interval

p: p value for **Odd`s ratio** for comparing between the studied groups.

**S-Table (6c): Univariate analysis for ROP (presence and absence) as regards blood samples, blood transfusion and B. Colostrum usage**

|  | | | **ROP** | | **OR (95% C.I)** | **P** |
| --- | --- | --- | --- | --- | --- | --- |
|  |  |  | **No** | **Yes** |  |  |
| **Blood samples** | | |  |  |  |  |
| Min. – Max. | | | 4.0 – 23.0 | 6.0 – 20.0 | 1.140 (1.027 – 1.265) | 0.014**^*^** |
| Mean ± SD. | | | 11.7 ± 4.5 | 14.3 ± 3.7 |  |  |
| Median (IQR) | | | 11.0  (8.0 – 15.0) | 15.0  (12.0 – 17.0) |  |  |
| **Blood transfusion** | | No | 76 (42.5%) | 1 (4.8%) | 1.000 |  |
|  |  | Yes | 103 (57.5%) | 20 (95.2%) | 14.757 (1.938 – 112.376) | 0.009**^*^** |
| **Number of blood transfusions** | | |  |  |  |  |
| Min. – Max. | | | 1.0 – 5.0 | 1.0 – 6.0 | 1.487 (1.023 – 2.163) | 0.038**^*^** |
| Mean ± SD. | | | 2.2 ± 1.2 | 2.8 ± 1.4 |  |  |
| Median (IQR) | | | 2.0 (1.0 – 3.0) | 2.5 (2.0 – 4.0) |  |  |
| **B. Colostrum** | No | | 84 (46.9%) | 16 (76.2%) | 1.000 |  |
|  | Yes | | 95 (53.1%) | 5 (23.8%) | 0.276 (0.097 – 0.787) | 0.016**^*^** |

OR: **Odd`s ratio** C.I: Confidence interval

p: p value for **Odd`s ratio** for comparing between the studied groups.

**S-Table (7): Comparison between the two studied groups as regards initial (D1) and day 14 (D14) laboratory data. ADD CRP**

|  | **Groups** | | **Test of sig.** | **P** |
| --- | --- | --- | --- | --- |
|  | **B. Colostrum** | **Control** |  |  |
| **HB-D1 (g/dl)** |  |  |  |  |
| Min. – Max. | 9.8 – 21.6 | 9.1 – 23.5 | t= -1.259 | 0.209 |
| Mean ± SD. | 15.6 ± 2.2 | 16.0 ± 2.3 |  |  |
| Median (IQR) | 15.7 (14.4 – 16.6) | 16.2 (14.6 – 17.5) |  |  |
| **Neutrophils-D1 (x103/dl)** |  |  |  |  |
| Min. – Max. | 1000.0 – 20000.0 | 300.0 – 30000.0 | U= 3660.5 | 0.001**^*^** |
| Mean ± SD. | 6536.0 ± 3642.1 | 5378.0 ± 4576.1 |  |  |
| Median (IQR) | 6050 (4000 – 9000) | 4100 (2750 – 6550) |  |  |
| **NLR-D1** |  |  |  |  |
| Min. – Max. | 0.3 – 7.0 | 0.3 – 11.0 | U= 3277 | <0.001**^*^** |
| Mean ± SD. | 1.7 ± 0.8 | 1.4 ± 1.2 |  |  |
| Median (IQR) | 1.7 (1.2 – 2.0) | 1.2 (0.8 – 1.8) |  |  |
| **PLT D1(x103/dl)** |  |  |  |  |
| Min. – Max. | 54.0 – 524.0 | 87.0 – 397.0 | t= -0.546 | 0.586 |
| Mean ± SD. | 218.4 ± 74.0 | 223.9 ± 68.7 |  |  |
| Median (IQR) | 213.5 (175.5 – 264.0) | 214 (173 – 280) |  |  |
| **PT D1(seconds)** |  |  |  |  |
| Min. – Max. | 10.0 – 39.0 | 11.3 – 31.0 | U= 4531.5 | 0.851 |
| Mean ± SD. | 16.7 ± 4.4 | 16.5 ± 4.0 |  |  |
| Median (IQR) | 16.0 (13.6 – 19.0) | 16.0 (13.4 – 19.0) |  |  |
| **PTT D1 (seconds)** |  |  |  |  |
| Min. – Max. | 35.0 – 120.0 | 41.0 – 150.0 | U= 4573 | 0.937 |
| Mean ± SD. | 63.9 ± 17.4 | 66.8 ± 24.1 |  |  |
| Median (IQR) | 61.0 (49.0 – 76.0) | 56.0 (50.0 – 80.0) |  |  |
| **PA -D1** |  |  |  |  |
| Min. – Max. | 17.0 – 100.0 | 24.0 – 107.0 | U= 4179.5 | 0.270 |
| Mean ± SD. | 57.0 ± 18.1 | 59.8 ± 18.5 |  |  |
| Median (IQR) | 52.0 (41.0 – 70.5) | 58.0 (46.0 – 74.0) |  |  |
| **INR -D1** |  |  |  |  |
| Min. – Max. | 0.8 – 4.0 | 0.9 – 2.9 | U= 4235.5 | 0.337 |
| Mean ± SD. | 1.6 ± 0.6 | 1.5 ± 0.4 |  |  |
| Median (IQR) | 1.5 (1.2 – 1.9) | 1.4 (1.2 – 1.7) |  |  |
| **CRP-D1 Ug/L** |  |  |  |  |
| Min. – Max. | 0.1 – 86.0 | 0.1 – 62.0 | U= 4532.5 | 0.253 |
| Mean ± SD. | 6.4 ± 11.2 | 6.1 ± 9.0 |  |  |
| Median (IQR) | 3.0 (0.5 – 7.0) | 3.3 (1.5 – 6.4) |  |  |
| **HB-D14** |  |  |  |  |
| Min. – Max. | 8.7 – 16.8 | 8.0 – 19.5 | U= 4056.5 | 0.021**^*^** |
| Mean ± SD. | 12.4 ± 1.8 | 11.8 ± 2.0 |  |  |
| Median (IQR) | 12.2 (11.0 – 13.9) | 11.7 (10.5 – 12.9) |  |  |
| **Neutrophils-D14** |  |  |  |  |
| Min. – Max. | 1600.0 – 24500.0 | 900.0 – 18900.0 | U= 4563 | 0.285 |
| Mean ± SD. | 5441.5 ± 3167.8 | 6248.0 ± 3729.5 |  |  |
| Median (IQR) | 5000 (4050 – 6000) | 5200 (3750 – 8100) |  |  |
| **NLR-D14** |  |  |  |  |
| Min. – Max. | 0.7 – 6.0 | 0.6 – 3.0 | U= 3697.5 | 0.001**^*^** |
| Mean ± SD. | 1.7 ± 0.7 | 1.4 ± 0.6 |  |  |
| Median (IQR) | 1.6 (1.3 – 1.8) | 1.3 (1.0 – 1.8) |  |  |
| **PLT-D14** |  |  |  |  |
| Min. – Max. | 147.0 – 675.0 | 100.0 – 705.0 | U= 4541.5 | 0.262 |
| Mean ± SD. | 321.1 ± 99.3 | 336.8 ± 116.4 |  |  |
| Median (IQR) | 318.0 (244.5 – 380.5) | 339.0 (249.5 – 411.5) |  |  |
| **PT-D14** |  |  |  |  |
| Min. – Max. | 11.0 – 17.0 | 11.5 – 20.0 | U= 1170 | 0.010**^*^** |
| Mean ± SD. | 13.6 ± 1.4 | 14.4 ± 1.7 |  |  |
| Median (IQR) | 13.2 (13.0 – 14.0) | 14.0 (13.0 – 15.6) |  |  |
| **PTT-D14** |  |  |  |  |
| Min. – Max. | 34.0 – 64.0 | 33.0 – 72.0 | U= 1240 | 0.031**^*^** |
| Mean ± SD. | 49.6 ± 4.7 | 53.2 ± 8.4 |  |  |
| Median (IQR) | 49.0 (48.0 – 52.0) | 51.0 (48.0 – 61.0) |  |  |
| **PA-D14** |  |  |  |  |
| Min. – Max. | 45.0 – 104.0 | 45.0 – 100.0 | U= 1278 | 0.053 |
| Mean ± SD. | 73.5 ± 8.7 | 70.5 ± 11.7 |  |  |
| Median (IQR) | 74.0 (69.0 – 78.0) | 69.0 (65.0 – 77.0) |  |  |
| **INR-D14** |  |  |  |  |
| Min. – Max. | 0.9 – 1.8 | 0.9 – 1.9 | U= 1256 | 0.036**^*^** |
| Mean ± SD. | 1.2 ± 0.2 | 1.3 ± 0.2 |  |  |
| Median (IQR) | 1.1 (1.1 – 1.3) | 1.2 (1.1 – 1.4) |  |  |
| **CRP-D14** |  |  |  |  |
| Min. – Max. | 0.1 – 58.0 | 0.1 – 94.0 | U= 3751 | 0.002**^*^** |
| Mean ± SD. | 4.4 ± 9.2 | 7.1 ± 13.9 |  |  |
| Median (IQR) | 1.5 (0.5 – 3.0) | 2.6 (1.2 – 4.9) |  |  |

t: Student t-test U: Mann Whitney test

p: p value for comparing between the studied groups *: Statistically significant at p ≤ 0.05

NLR: Neutrophil to lymphocyte ratio

S-table 8: Nutritional composition of PreNAN formula( per 100 gram powder and per 100 ml of prepared formula).NB. Image of composition on the can is shown in the lower pannel.

| Composition | Per 100-gram powder | Per 100 ml prepared formula |
| --- | --- | --- |
| Energy | 498 | 80 |
| Protein (g) | 14.4 | 2.32 |
| Fat (g) | 25.9 | 4.17 |
| Carbohydrate (g) | 53.2 | 8.5 |
| Minerals (Ash)(g) | 3.5 | 0.56 |
| Sodium (mg) | 269 | 43 |
| Potassium (mg) | 605 | 97 |
| Chloride (mg) | 430 | 69 |
| Calcium (mg) | 760 | 122 |
| Phosphorus (mg) | 445 | 71.5 |


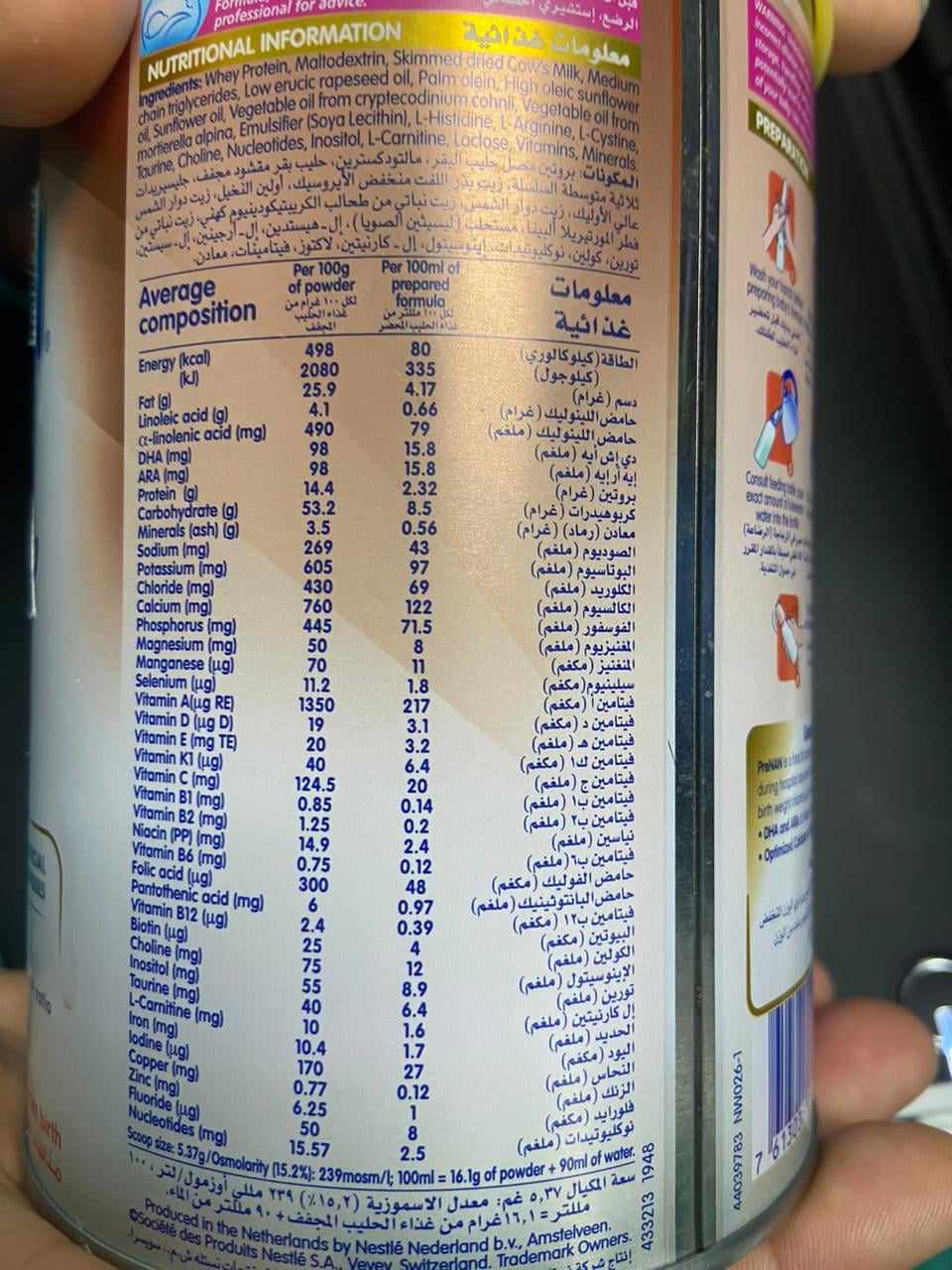


S-table9: Nutritional composition of BC (baby steps)


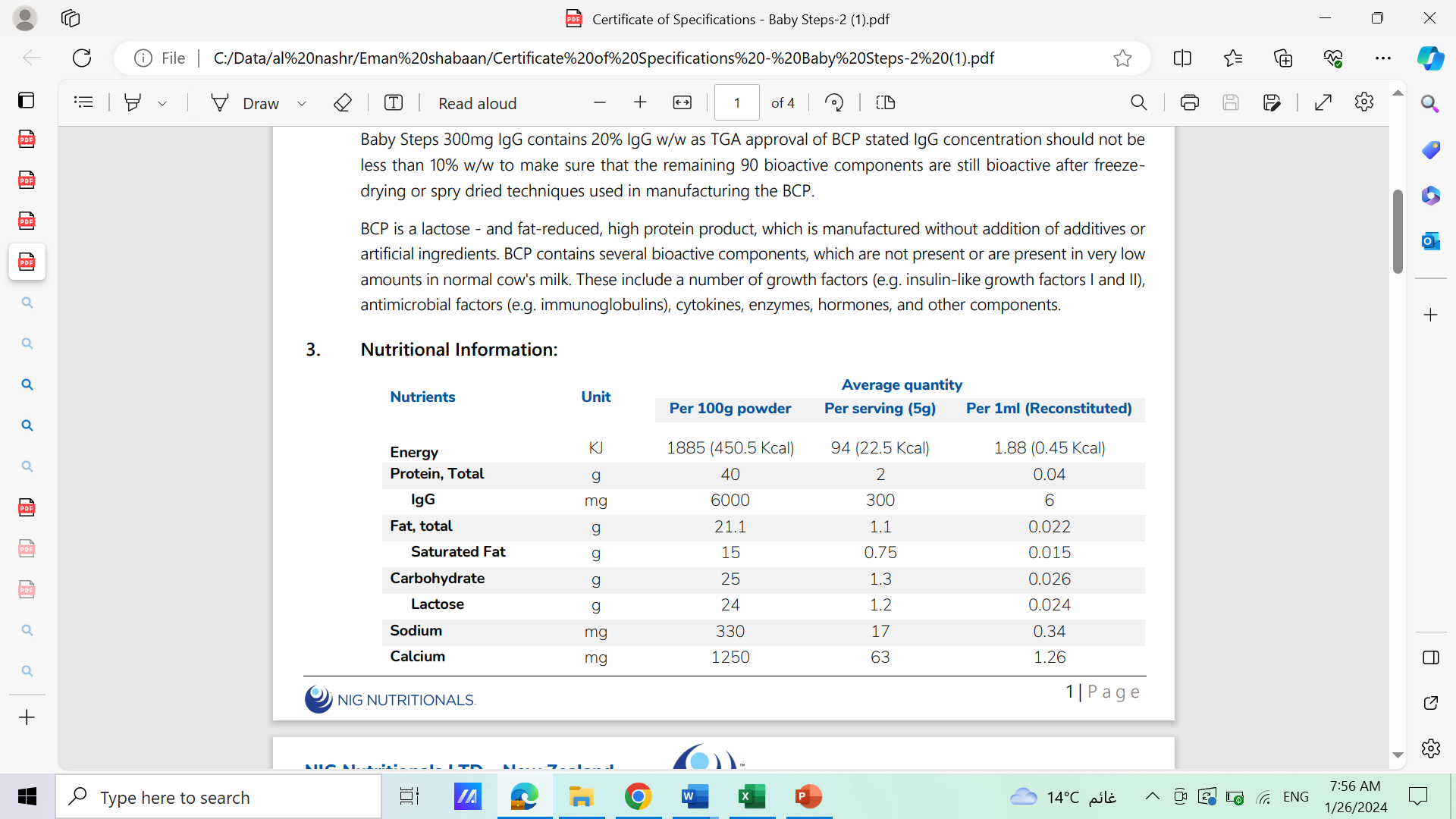

Supplement: Supplementary file 1 — Supplementary Material 1 [file 13052_2024_1781_MOESM1_ESM.docx]
